# The Therapeutic Potential of MicroRNA-21 in the Treatment of Spinal Cord Injury

**DOI:** 10.3390/cimb47020070

**Published:** 2025-01-21

**Authors:** Ahmed Hasan, Alessio Ardizzone, Domenico Giosa, Sarah Adriana Scuderi, Elsa Calcaterra, Emanuela Esposito, Anna Paola Capra

**Affiliations:** 1 Department of Chemical, Biological, Pharmaceutical and Environmental Sciences, University of Messina, Viale Ferdinando Stagno D’Alcontres, 31, 98166 Messina, Italy; ahmed.hasan@unicam.it (A.H.); aleardizzone@unime.it (A.A.); domenico.giosa@unime.it (D.G.); sarahadriana.scuderi@unime.it (S.A.S.); elsa.calcaterra@studenti.unime.it (E.C.); annapaola.capra@unime.it (A.P.C.); 2 Center of Neuroscience, School of Advanced Studies, University of Camerino, 62032 Camerino, Italy

**Keywords:** spinal cord injury (SCI), neurotraumas, microRNA, miR-21, inflammation, apoptosis

## Abstract

Spinal cord injury (SCI) involves complex pathological processes that often result in significant and long-term neurological deficits. Increasingly, research has identified microRNA-21 (miR-21) as a pivotal regulator in SCI, with studies focusing on its roles in inflammation, apoptosis, and tissue repair. This review synthesizes current findings on miR-21’s involvement in post-injury molecular events, emphasizing its interactions with regulatory targets such as Phosphatase and Tensin Homolog (PTEN) and Programmed Cell Death Protein 4 (PDCD4), as well as its broader effects on inflammatory and apoptotic signaling pathways. Evidence from both in vitro and in vivo studies suggests that modulating miR-21 influences lesion size, cellular dynamics, and functional recovery, highlighting its potential as a therapeutic target for SCI. Nonetheless, the clinical translation of miR-21-based therapies poses significant challenges, including the need to optimize dosages, delivery mechanisms, and long-term safety profiles. Further research is crucial to fully delineate miR-21’s therapeutic potential and determine its feasibility for integration into SCI treatment protocols. This review aims to provide a comprehensive overview of miR-21’s roles in SCI pathology, offering insights into the molecular mechanisms underlying recovery and the emerging potential of miR-21 in SCI management to enhance outcomes and quality of life for affected patients.

## 1. Introduction

Spinal cord injury (SCI) is a debilitating condition that results from damage to the spinal cord, often leading to varying degrees of loss of sensory and motor function [1]. This injury can be classified into two main categories: complete and incomplete. Complete SCI results in a total loss of function below the level of injury, while incomplete SCI allows for some residual function [2]. The causes of SCI are diverse, including traumatic events such as vehicle accidents, falls, sports injuries, and violence, as well as non-traumatic factors like tumors, infections, or degenerative diseases [3].

The impact of SCI is profound, not only affecting physical capabilities but also significantly altering psychological and social aspects of an individual’s life [4]. Patients often face challenges such as paralysis, loss of bowel and bladder control, respiratory issues, and chronic pain [5]. The severity and location of the injury dictate the specific symptoms and the extent of functional impairment, which can range from limited mobility to complete paralysis (quadriplegia or paraplegia) [6]. Rehabilitation is a critical component of the recovery process, aimed at maximizing functional independence and quality of life, this may involve physical therapy, occupational therapy, and various assistive technologies [7]. Recent advancements in research have also explored potential therapeutic avenues, including stem cell therapy, neuroprotective agents, and gene therapy, intending to promote spinal cord regeneration and functional recovery [8]. Psychological support is equally essential, as individuals coping with SCI often experience anxiety, depression, and social isolation, thus, multidisciplinary care should include physical, emotional, and social support to enhance recovery outcomes [4].

Current therapies for SCI focus on immediate stabilization, preventing secondary damage, and maximizing recovery through rehabilitation and supportive care [9]. Initial emergency care includes spinal immobilization and, if necessary, surgical intervention to stabilize the spine and relieve pressure on the spinal cord [9].

Pharmacologically, corticosteroids like methylprednisolone are administered shortly after injury to reduce inflammation, while neuroprotective agents are being explored to inhibit nerve cell damage [10]. Pain management is also crucial, employing analgesics and treatments for neuropathic pain [11]. Despite advancements in therapies for SCI, significant limitations remain, underscoring the need to explore alternative compounds and treatment strategies [12]. Indeed, one major limitation is the timing of corticosteroids, such as methylprednisolone, which must be administered within a narrow time window after injury to be effective [13]. However, their use is controversial due to potential side effects, including infection risk and limited long-term benefits [14]. Additionally, while neuroprotective agents show promise, many are still in experimental stages, and their clinical application remains limited due to a lack of robust evidence supporting their efficacy in human subjects [14].

Differently, rehabilitation of SCI incorporates physical therapy to improve strength and mobility, occupational therapy to assist in daily living activities, and the use of assistive devices like wheelchairs and braces to enhance independence [15].

Regarding this, rehabilitation therapies, though essential, often yield variable outcomes, heavily dependent on the severity of the injury and the individual’s response to treatment [15]. Some patients may experience limited improvement despite intensive rehabilitation, emphasizing the need for more effective therapeutic options [15]. Furthermore, pain management can be challenging, as standard analgesics may not adequately address neuropathic pain, and long-term use of opioids raises concerns about dependency and side effects [16]. These limitations underscore the urgent need to explore new compounds and innovative therapeutic strategies. Research into stem cell therapies, biomaterials, and molecular approaches, such as gene therapy and the use of growth factors, holds promise for enhancing recovery and promoting regeneration in the injured spinal cord [17]. By probing these alternative avenues, the hope is to develop more effective treatments that can significantly improve functional outcomes life for individuals with SCI.

MicroRNAs (miRNAs) hold significant potential as therapeutic compounds in the treatment of various conditions, including SCI. These small, non-coding RNA molecules play a crucial role in regulating gene expression by binding to messenger RNAs (mRNAs) and inhibiting their translation or promoting their degradation [18]. This regulatory capability positions miRNAs as powerful modulators of cellular processes, including inflammation, apoptosis, and neuronal regeneration, which are critical factors in the recovery from SCI [19].

One of the key advantages of miRNAs is their ability to target multiple genes simultaneously, allowing for a more comprehensive approach to therapy. For instance, specific miRNAs can modulate pathways involved in neuroprotection, neuroinflammation, and tissue repair, potentially enhancing the body’s intrinsic repair mechanisms following injury [20]. Furthermore, miRNAs can be delivered using various methods, including viral vectors, nanoparticles, and exosomes, providing flexibility in therapeutic design [21].

In the context of SCI, studies have demonstrated that manipulating the expression of certain miRNAs can promote neuronal survival, enhance axonal regeneration, and reduce glial scarring, which are essential for functional recovery [22]. Additionally, miRNA therapy could be used to suppress deleterious miRNAs that contribute to injury progression, thereby fostering an environment conducive to recovery [23].

Nevertheless, despite their promise, the clinical application of miRNAs faces challenges, including the need for precise delivery mechanisms, understanding the long-term effects of miRNA modulation, and potential off-target effects [24]. Ongoing research is focused on these challenges to refine miRNA-based therapies and explore their synergistic effects with other treatments.

In this broad context, our attention focused on microRNA-21 (miR-21), which was shown to play a protective role in neuronal cells, promoting survival and reducing inflammation after brain injuries [25,26].

Therefore, based on all these assumptions, this review aimed to assess the therapeutic potential of miR-21 within the context of SCI, to better validate its neuroprotective role.

## 2. Pathophysiology of SCI

The pathophysiology of SCI can be broadly categorized into two phases: the primary injury phase, involving the initial traumatic event, and the secondary injury phase, which includes a cascade of biochemical and cellular changes exacerbating the initial damage.

### 2.1. Primary Injury Phase

The primary injury phase of SCI occurs at the moment of trauma and is caused by mechanical forces directly impacting the spinal cord [2]. This phase typically involves compression, contusion, or shear forces that disrupt the spinal cord’s anatomical structure [2]. These forces can fracture vertebrae or dislocate them, leading to immediate compression of the spinal cord and damage to the neurons, axons, and blood vessels. This structural damage causes cell membrane rupture, leading to the release of intracellular ions and neurotransmitters into the extracellular space, triggering a rapid inflammatory response [2].

Hemorrhage is another hallmark of the primary injury phase, as blood vessels rupture within the spinal cord, creating localized bleeding [27]. This causes an initial loss of blood flow, or ischemia, which deprives the tissue of essential oxygen and nutrients, further aggravating cellular damage [27]. In turn, these effects initiate a cascade of cellular death pathways and prepare the tissue environment for the secondary injury phase, where inflammation, excitotoxicity, and other biochemical processes exacerbate initial trauma [28]. Although the primary injury phase is irreversible, understanding its mechanisms is crucial for developing interventions to mitigate further damage during the subsequent phases of SCI.

### 2.2. Secondary Injury Phase

The secondary injury phase in SCI begins shortly after the initial trauma and can continue over hours to weeks [28]. This phase involves a series of complex biochemical and cellular events that further damage the spinal tissue and significantly influence the severity of functional deficits, indeed, key mechanisms in the secondary injury phase include ischemia, inflammation, excitotoxicity, oxidative stress, and apoptosis [29].

Ischemia occurs due to damaged blood vessels and compromised blood flow around the injury site, this reduces the oxygen and nutrient supply, worsening neuronal and glial cell damage [29]. Inflammation soon follows, with immune cells such as neutrophils and macrophages infiltrating the injured area and releasing pro-inflammatory cytokines like tumor necrosis factor-α (TNF-α) and interleukins [30]. Although initially aimed at tissue repair, prolonged inflammation often leads to additional injury by exacerbating oxidative stress and excitotoxicity [30].

Excitotoxicity is another critical process in secondary SCI, driven by the excessive release of glutamate from damaged neurons [31]. This leads to the overstimulation of NMDA (N-methyl-D-aspartate) and AMPA (alpha-amino-3-hydroxy-5-methyl-4-isooxazole-propionic acid) receptors on nearby neurons, causing an influx of calcium ions, which activate enzymes that degrade cell structures and lead to further cell death [32].

Furthermore, oxidative stress, triggered by the accumulation of reactive oxygen species (ROS) and reactive nitrogen species (RNS), damages lipids, proteins, and DNA within cells [33]. This stress overwhelms the cells’ natural antioxidant defenses, contributing to mitochondrial dysfunction and subsequent cell death [34].

As the secondary injury progresses, apoptosis or programmed cell death occurs in neurons, oligodendrocytes, and astrocytes [35]. This further impacts the structural and functional integrity of the spinal cord, with oligodendrocyte loss leading to demyelination and impaired signal conduction [35]. Another result of this phase is glial scar formation, in which astrocytes proliferate and form a dense scar around the injury site [36]. While glial scarring helps contain inflammation, it also creates a physical and chemical barrier that inhibits axonal regeneration, complicating recovery [36].

The secondary injury phase thus transforms the initial trauma into more extensive damage, highlighting the importance of timely interventions. Therapies that can mitigate oxidative stress, reduce inflammation, or prevent excitotoxicity during this phase hold the potential to improve SCI outcomes by limiting additional cellular damage and supporting tissue preservation [37].

## 3. Role of miRNAs in SCI

MiRNAs are small, non-coding RNA molecules that play critical roles in regulating gene expression and influencing various biological processes, including SCI [38]. As stated, following SCI, a cascade of molecular events occurs, during which specific miRNAs are dysregulated, significantly impacting inflammation, apoptosis, and neuronal regeneration [39]. For instance, miR-146a plays an essential role in attenuating inflammation post-injury by targeting nuclear factor-kappa B (NF-κB), a crucial factor in cytokine production, thereby contributing to a reduced inflammatory response [40]. Conversely, miR-155 can exacerbate inflammation by promoting pro-inflammatory cytokine expression, highlighting the necessity for a balanced miRNA profile for optimal recovery [41].

Beyond inflammation, miRNAs also regulate apoptosis, a pivotal determinant of neuronal survival after injury, in fact, specific miRNAs are known to inhibit pro-apoptotic factors, thereby promoting cell survival and maintaining tissue integrity. For example, miR-133b has been demonstrated to inhibit apoptosis in neurons following SCI [42,43], thereby enhancing neuronal resilience. Furthermore, miR-124 and miR-9 have been identified as crucial for neuroprotection and regeneration, as they enhance neuronal differentiation and promote axonal outgrowth, facilitating recovery of motor and sensory functions [44,45].

Glial scarring is another critical response to SCI that serves both protective and detrimental roles. The formation of glial scars by reactive astrocytes is influenced by various miRNAs, including miR-145, which can modulate astrocyte activation and proliferation [46,47]. While glial scarring helps limit inflammation and stabilize the injury site, it also creates a physical barrier that obstructs axonal regeneration [48]. Studies have shown that manipulating the expression of specific miRNAs can alter the extent of glial scarring and influence neuronal survival [49,50].

Given these complexities, targeting miRNAs presents a promising therapeutic strategy for enhancing recovery post-SCI. Current research is exploring methods to deliver miRNA mimics or inhibitors through various delivery systems, such as nanoparticles, viral vectors, and exosomes, to achieve targeted and sustained delivery to the injury site [51]. For example, studies have demonstrated that miR-219 has been found to promote oligodendrocyte differentiation, which is critical for remyelination after injury, suggesting that enhancing its expression may support functional recovery [52].

While the therapeutic potential of miRNAs in SCI is significant, challenges remain regarding effective delivery and off-target effects. Ongoing studies aim to optimize these delivery systems, which may improve localized and sustained release of miRNAs, thereby enhancing therapeutic efficacy while minimizing systemic side effects. Furthermore, exploring the synergistic effects of combining different miRNA therapies may yield more effective recovery strategies.

Overall, the complex roles of miRNAs in SCI emphasize their importance as therapeutic targets. Among these, miR-21 stands out as a particularly significant factor in mediating responses to injury and holds promise for future therapeutic strategies aimed at improving recovery and functional outcomes in individuals suffering from SCI. Continued investigation into the specific mechanisms and pathways influenced by miR-21 will be essential for developing innovative miRNA-based therapies that could transform the clinical management of SCI.

## 4. MiR-21

### 4.1. Structure and Function of miR-21

MicroRNAs have been identified as a potential regulator of gene expression in numerous processes related to the development and progression of diseases, involving the process of proliferation, development, and cellular immunity [53]. MiR-21 has already been detected in human blood cell lines including macrophages and neutrophils [54]. Studies demonstrate evidence of its important role in the regulatory function and its potential as a biomarker in SCI [55]. Exploring the direct interaction of miR-21 with the target gene makes it possible to establish a new therapeutic approach by controlling the expression of the specific target gene.

### 4.2. Characteristics of miR-21

MiR-21 is a single-stranded, non-coding RNA molecule of 22 nucleotides long. The miR-21 coding sequence is located on chromosome 17q23.2 in the 10 intronic region of the gene coding for transmembrane protein 49 (TMEM49). Although the two genes overlap, both are transcribed in the same direction but from different promoters, because their expression is not correlated [56]. MiR-21 originates from a longer primary transcript pri-miR-21, which is processed to form the mature miR-21 from two steps. Initially, pri-miR-21 begins to be processed in the nucleus after its transcription, where it is cleaved by the enzymatic action of Drosha and DGCR8, forming the pre-miR-21 [57,58]. The pre-miR-21 is then transported to the cytoplasm by the action of Exportin-5 to continue its maturation process. In the cytoplasm, it undergoes cleavage by the action of the Dicer enzyme, which cuts the loop region, forming a double-strand miR-21 with two single strands. This miR-21 then interacts with the RNA-induced silencing complex (RISC) leading to the formation of the final functional form of the miRISC complex, which binds to the 3′ untranslated region (UTR) of the target mRNA to modulate its expression [59]. After their formation, miR-21 begins to act on the target, promoting the regulation of gene expression and protein production, and thus can play important regulatory roles in the organism.

### 4.3. Expression Pattern of miR-21 in SCI

MiR-21 has been described as an important miRNA for the physiology of central nervous system disorders including SCI and plays an essential function in the controlling of gene expression following SCI [55]. The overexpression of miR-21 in SCI has been shown in several studies, suggesting its involvement in this disorder. Wang et al. found that the expression level of miR-21-5p was increased after SCI [60]. In contrast, animals with miR-21-5p knockdown presented a significant improvement in functional recovery after SCI along with the reduced profile of fibrotic scar formation and improved axonal regeneration. MiR-21-5p contributed to enhancing the circuit amplification of the transforming growth factor-β (TGF-β) signaling pathway by targeting mothers against decapentaplegic homolog 7 (Smad7) resulting in fibrosis formation. The study suggests miR-21-P as a potential target for the treatment of SCI [60]. In vitro, overexpression of miR-21a-5p after mechanical injury of primary spinal fibroblast was observed, this expression promoted fibrogenic activity by enhancing Smad2/3 phosphorylation leading to increased proliferation and decreased apoptosis [61].

MiR-21 was found overexpressed in the different time points starting from the first time point 4 h and reached the maximum at one week in both severe and mild injuries [62]. In the C57BL/6 mice model of SCI, it was revealed that miR-21 is overexpressed in spinal cord tissue around the injury in comparison to healthy tissue. The knockdown of miR-21 suppressed inflammation, improved neuronal recovery, and promoted neuronal regeneration [63]. In another in vitro study conducted on rat spinal cord neurons (RN-sc), the cells were stressed by H_2_O_2_. The outcomes demonstrated that the miR-21 was upregulated after stress induced by H_2_O_2_ while silencing miR-21 reduced H_2_O_2_-induced cell death and apoptosis [64]. However, other research has shown a rise in miR-21 expression after SCI, which plays a crucial role in improving functional recovery and enhancing neuronal survival in rats. In PC-12 cells with oxygen-glucose deprivation (OGD), miR-21 expression is significantly increased, which leads to a decrease in apoptosis by inhibiting the programmed cell death protein 4 (PDCD4)/Caspase-3 (Casp-3) pathway [65]. In another study, the overexpression of miR-21 in vivo was achieved through intrathecal injection of lentivirus vectors containing pre-miR-21 [66]. Transfection of pre-miR-21 significantly enhanced expression of miR-21 in the spinal cord and significantly downregulated expressions of Casp-3, Fas ligand (FasL), and PDCD4 by inhibition of the proapoptotic proteins of FasL and PDCD4 [66]. Astrocyte polarization contributes to determining the outcome of SCI with the emergence of miR-21 as a key regulator in this process [67]. It was demonstrated that miR-21 levels increase in a time-dependent manner following SCI in mice. High expression of miR-21 in astrocytes near the lesion site was shown to reduce the hypertrophic response to SCI. Conversely, inhibiting miR-21 function led to an increased hypertrophic phenotype in astrocytes, resulting in greater axon density within the lesion site [68].

Building on this, another study revealed that miR-21a-5p significantly promotes the transformation of naïve astrocytes into neurotoxic reactive astrocytes (A1s) following traumatic spinal cord injury (TSCI) by downregulating Ciliary Neurotrophic Factor Receptor α (CNTFRα) [69]. This mechanism enhances the inflammatory response associated with SCI, as evidenced by increased A1 marker expression and decreased A2 markers upon miR-21a-5p modulation. Additionally, CNTF treatment was shown to inhibit A1 polarization through the activation of the Signal Transducer and Activator of Transcription-3 (STAT3) signaling pathway, suggesting a potential therapeutic approach for mitigating inflammation [69].

In contrast, silencing miR-21 was shown to promote the polarization of astrocytes to the neuroprotective A2 phenotype following acute ischemic spinal cord injury (ISCI). This polarization enhances synapse formation and nerve growth by targeting glypican 6 through the STAT3 pathway. Both in vivo and in vitro experiments confirmed that ischemic conditions transform naïve astrocytes into A2 phenotypes, underscoring the therapeutic potential of targeting miR-21 in SCI recovery [70].

In a study conducted on 52 C57BL/6 male mice with moderate contusion, the authors found that miR-21 was significantly upregulated in astrocytes after SCI, especially in areas associated with glial scar formation. This upregulation suggests that miR-21 may positively influence the recovery process after SCI by facilitating the modulation of the TGF-β signaling pathway, which is known to influence astrocytic behavior and scar formation [71]. In inflamed spinal cord tissues, miR-21 expression increases significantly post-injury, contributing to the activation of the Interleukin-6-receptor (IL-6R)/JAK-STAT pathway, which exacerbates inflammation. In activated microglial cells, the administration of a miR-21 inhibitor effectively reduces the expression of inflammatory factors such as inducible nitric oxide synthase (iNOS) and TNF-α, suggesting that miR-21 promotes inflammation. The research also found that the use of miR-21 inhibitors reduces RNA levels of IL-6R, JAK, and STAT3, which are part of the inflammatory signaling pathway [72].

Jiang et al. investigated the effect of miR-21 on PDCD4 and PTEN using 150 female Sprague Dawley rats. They revealed that the expression level of miR-21 is significantly downregulated in injured spinal cords at 4 and 8 h, and 1 day post-injury, but shows a marked increase at 3 and 7 days post-injury [73]. MiR-21 promotes neurite outgrowth by downregulating PDCD4 and enhancing axonal regeneration following SCI [73]. miR-21 was found to be prominently expressed in areas of neuronal damage 1 day after contusion SCI, with continued dysregulation observed at 3 days post-injury, indicating its potential role in the injury response. The study utilized miRNA microarray analysis to identify these expression changes, revealing that miR-21 upregulation correlates with functional recovery and neuronal survival. Furthermore, the knockdown of miR-21 exacerbated functional deficits and increased apoptotic cell death [74].

On the contrary, it was found that miR-21 expression decreased in SCI animals compared to sham-operated controls, while PTEN and PDCD4 levels increased. MiR-21 derived from mesenchymal stem cell (MSC) exosomes containing miR-21 improved functional recovery by reducing neuronal death through targeting Casp-3, PTEN, and PDCD4, presenting potential as a therapeutic target in cell-based therapies for SCI [75].

Collectively, the expression levels of miR-21 show variation in the different models of SCI, likely due to the different models used, the timing of detection after the injury, and the cell type involved in the experimental design, especially in vitro studies. Consequently, it is required to conduct more in vivo studies on miR-21 as a potential SCI diagnostic biomarker. However, the vast majority of studies indicated that the overexpression of miR-21 following SCI may provide protective effects on neurons through various mechanisms, suggesting it could serve as a novel therapeutic target as shown in Table 1.

Focusing exclusively on the in vivo studies, we examined the differences in the SCI model protocols, the vertebral level at which the injury was induced, as well as the dose and duration of the various treatments related to miR-21, as summarized in Table 2.

### 4.4. MiR-21 in Inflammation and Immune Response During SCI

The immune system often requires rapid activation or inhibition, involving reactive and efficient regulation of mRNA expression generated in part by the action of microRNAs. During the immune response, several cells intervene such as T lymphocytes, which by proliferating and developing, change from naïve T cells to effector T cells responsible for the adaptive immune response. Once the pathogen or the damage is eliminated, effector T lymphocytes are massively destroyed or transformed into memory T lymphocytes [76]. The expression levels of proteins and miRNAs in these three types of T lymphocytes vary, reflecting the different functions of each cell. miR-21 has been identified in the maintenance of the effector phase of T cells. Indeed, its expression level is high in effector T lymphocytes, decreased in memory T lymphocytes, and very low in naïve T lymphocytes [77]. In inflammatory diseases like asthma induced by interleukin-13 (IL-13) or allergens, miR-21 is overexpressed in monocyte and macrophage lineage cells. The increase in miR-21 levels is reflected by a decrease in the level of the cytokine IL-12 involved in the adaptive immune response by T helper 1 (Th1) against intracellular bacteria. Indeed, IL-12p35 mRNA contains in its 3’-UTR a potential target sequence of miR-21 which, upon binding, inhibits the translation of the mRNA, thus promoting the immune response made by Th2 lymphocytes against extracellular pathogens [78]. Regulation of the level of miR-21 thus makes it possible to adapt to the immune response to the different stresses encountered. In the context of SCI, the elevation of miR-21 is significant. A clinical study of 24 patients with SCI and 24 healthy people found that the expression level of miR-21-5p was significantly elevated in the serum of SCI patients, and the expression of gene Pleomorphic Adenoma Gene 1 (*PLAG1*) was downregulated, suggesting the correlation between them, alongside inflammatory factors like TNF-α, IL-1, and IL-6, was elevated in SCI patients [79]. SCI can activate the release of different secretory factors including cytokines that stimulate immune responses, which attach to cell surface receptors and stimulate immune responses by excessive inflammation of Th1 and Th17 and microglia activation. This pro-inflammatory response enhances the signaling pathways like NF-κB, which act as transcription factors for miR-21 and lead to an induction in the context of inflammatory processes [80]. This delayed induction indicates a regulatory function of miR-21 in inflammatory reactions in the sense of a feedback mechanism [81,82]. Although miR-21 shows inflammatory roles, it is the predominant anti-inflammatory miR in the nervous system and has the potential to regulate neuroinflammation [83]. Previous studies found an increase in the release of pro-inflammatory cytokines TNF-α and IL-1β 14 days after SCI, which act on T lymphocytes to enhance scar formation. However, these pro-inflammatory cytokines dramatically decreased in the group that received miR-21 inhibitors [63,80]. A study with human peripheral blood demonstrated the ability of miR-21 to participate in the differentiation of T cells. The decrease in its expression favored the increase in memory cells, as it is correlated with genes responsible for the production of these cells [84]. MiR-21 is considered one of the most frequently dysregulated miRNAs in inflammatory diseases [81] contributing to various immune and inflammatory responses [85].

Conversely, miR-21 overexpression following SCI has been linked to a reduction in inflammatory processes as it modulates the PI3K/AKT pathway and suppresses inflammatory cytokines such as IL-6, IL-1β, IL-8, endothelial nitric oxide synthase (eNOS), and TNF-α [86]. Furthermore, miR-21 binds to the 3′UTR region of IL-1β mRNA, inhibiting its expression and subsequent protein production [86].

MiR-21 has probable targets related to the innate immune system. A correlation was observed between the levels of miR-21 and those of chemokine C-C motif ligand 3 (CCL3), in a neonatal rat ischemic model, in which miR-21 exhibited a suppressive activity on the proinflammatory chemokine CCL3 [87]. CCL3 is a chemokine released by macrophages activated via Toll-like receptors (TLR), which can contribute to inflammation and tissue damage after SCI. The expression levels of CCL3 and its receptor have been increased in animal models with SCI, suggesting their role in the inflammatory response and tissue damage [88].

**Figure 1:**
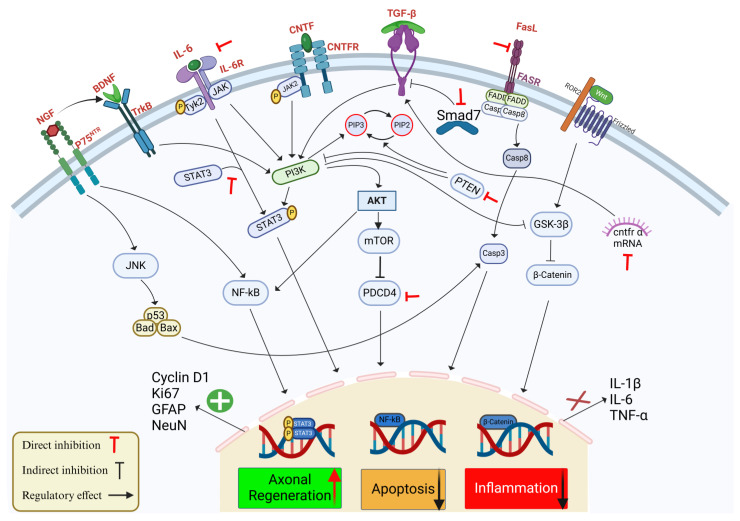
Schematic representation of the effects of miR-21 on key signaling pathways involved in SCI, with a focus on axonal regeneration, apoptosis, and inflammation. MiR-21 regulates multiple pathways responding to SCI by targeting multiple components that influence cellular outcomes. The interaction between neurotrophic factors, e.g., BDNF and nerve growth factor (NGF) and their receptors tropomyosin receptor kinase B (TrkB) and P75 activate downstream signaling pathways, such as c-Jun N-terminal Kinase (JNK) and NF-ĸB, contributing to cell survival and regeneration. MiR-21 regulates axonal regeneration by promoting STAT3 phosphorylation and stimulating the expression of regeneration markers: Cyclin D1, Ki67, Glial Fibrillary Acidic Protein (GFAP), and Neuronal Nuclei (NeuN). In parallel, miR-21 decreases apoptosis through the Phosphoinositide 3-Kinase (PI3K)/AKT pathway, which downregulates pro-apoptotic factors, e.g., Casp-3 and Caspase-8 (Casp-8), and by the direct inhibition of PTEN and PDCD4, which provides neuronal protection and enhance survival. Inflammatory responses mediated by IL-1β, IL-6, and TNF-α are attenuated by miR-21 interacting with Transforming Growth Factor-β (TGF-β)/Smad7 signaling and suppressing NF-ĸB, thereby reducing inflammation. In addition, miR-21 modulates the FasL/Fas Receptor (FASR) axis and the Wnt/β-catenin pathway to mitigate cell death further and promote neural repair. Red inhibitory arrows represent the direct inhibition by miR-21, black inhibitory arrows represent the indirect effect of miR-12 on the signaling pathway, and enhancing arrows indicate the regulatory effects of miR-21 on these pathways.

Although research is limited on the interaction of nitric oxide (NO) and ROS with miR-21 in the pathology of SCI, it has been demonstrated that in human endothelial cells, the expression of miR-21 leads to an increased NO synthesis and inhibition of apoptosis [89]. However, in microglia, the expression of inducible nitric oxide synthase (iNOS) in vitro was depressed after inhibition of miR-21 [72]. Furthermore, miR-21 can also regulate the expression of Krev Interaction Trapped 1 (*KRIT1)*, which is involved in maintaining ROS homeostasis [90] and the expression of the Superoxide Dismutase 3 (*SOD3*) gene, which also modulates ROS levels in human epithelial cells [91]. Therefore, miR-21 acts in the regulation of NO and ROS in the immune response; however, a better understanding of this mechanism in SCI is necessary to develop therapies targeting miRNAs. The main signaling pathways affected by miR-21 involved in SCI are summarized in Figure 1.

### 4.5. Link Between miR-21 and Apoptosis in SCI

MiR-21 plays a significant role in apoptosis after SCI; indeed it directly targets several mRNAs transcribed from pro-apoptotic genes such as PDCD4, PTEN, FasL, or Bcl-2 by regulating their expression level, thus suggesting that miR-21 overexpression after SCI may exert protective effects [92].

Hu et al. found that the antagonism of endogenous miR-21 in rats after SCI leads to a weakened neurological recovery, an increase in the size of the spinal cord lesion, and an increase in apoptosis [74]. In PC-12 cells, overexpressed miR-21 targets and inhibits PDCD4 and influences Casp-3 activity, which finally leads to an anti-apoptotic effect and reduced neuronal cell death [65]. Another study in vitro illustrated that miR-21 affects the expression of key apoptotic proteins. It was found that miR-21 promotes the expression of Bcl-2 and Proliferating Cell Nuclear Antigen (PCNA) proteins while inhibiting the expression of Bax and Casp-3. This balance is crucial for cell survival and indicates that miR-21 has a protective role against apoptosis in astrocytes [71]. Another research study has demonstrated that upregulation of miR-21 level can improve neuronal survival in both vivo and in vitro SCI models. It was illustrated that miR-21 exerts anti-apoptotic effects by targeting genes such as PDCD4 and PTEN, leading to reduced neuronal cells and providing potential therapeutic avenues for recovery from SCI [75,92]. In a study conducted by Jiang et al., the expression of miR-21 was significantly altered following SCI, with levels initially decreasing and then increasing several days after the injury [73].

Notably, miR-21 overexpression in cultured spinal cord neurons promoted neurite outgrowth while downregulating the expression of its target gene, PDCD4, without affecting PTEN levels [73]. The luciferase reporter assays confirmed that miR-21 directly targets the 3′ untranslated region of PDCD4 mRNA, leading to decreased protein expression. Additionally, the study found that increased miR-21 levels may create a more favorable environment for nerve regeneration by inhibiting pro-apoptotic factors [73].

Overall, lower apoptosis and better recovery results have been associated with miR-21 overexpression. However, it must be taken into consideration that the expression of miR-21 differs between SCI models, as they could be influenced by the model used and the time of detection post-lesion.

### 4.6. MiR-21 in Cell Survival and Axonal Regeneration After Spinal Trauma

After SCI, the overexpression of miR-21 influenced the differentiation of the neural stem cells (NSCs) into oligodendrocytes, which regulate the axonal remyelination process. MiR-21 upregulation targets Smad7 and activates the TGF-β/Smad2 signaling pathway, which promotes glial scar formation and impaired remyelination; it was found that inhibition of miR-21 reverses these processes [93]. Another mechanistic effect of miR-21 upregulation on the differentiation of NSCs is increased cell proliferation through activation of cyclin D1 expression, associated with an increase in activation of the PI3K/AKT signaling pathway [94]. MiR-21 overexpression plays a significant role in promoting the differentiation and proliferation of NSCs by increasing the activation of AKT/GSK-3β signaling pathways [95]. On the other hand, knocking down miR-21 decreases NSC differentiation by preventing cyclin D1 expression [95].

Moreover, miR-21 knockdown inhibits cyclin D1 expression and interferes with the AKT/GSK-3β signaling pathway, which in turn prevents the differentiation of neural stem cells [95]. Beyond these pathways, the Wnt/β-catenin pathway has also been shown to play a crucial role in determining the fate of NSCs. Activating this pathway has been associated with an increase in the proliferation and differentiation of NSCs [96]. Accordingly, miR-21 can boost the growth of neural stem cells and facilitate their transformation into neurons, while simultaneously reducing their differentiation into astrocytes through the Wnt/β-catenin signaling pathway [96].

### 4.7. The Importance of Different Preclinical Models in SCI Research

Preclinical studies related to miR-21 in spinal cord injuries involve the use of various models that aid in understanding the different aspects related to spinal cord injuries, according to their treatments. Traumatic injuries caused by controlled mechanical impact on the spinal cord closely mimic human spinal cord injuries in terms of inflammation, ischemia, and lesion formation [97,98]. On the other hand, transection models, which involve a complete severing of the spinal cord either partially or completely, are more effective for studying regeneration and functional recovery [97,98].

The timing of injuries also significantly affects the experiments. Studies in the acute phase last hours to days post-injury and mainly focus on inflammation, oxidative stress, and programmed cell death to manage the immediate cellular and molecular reactions and early treatment measures [99]. In contrast, studies in the chronic phase last weeks to months post-injury focus on axonal growth and functional recovery. Research in acute traumatic injury models has demonstrated reduced inflammation and scar formation in the context of miR-21 [75]. Similarly, studies in the chronic phase have shown that miR-21 plays a role in promoting axonal growth and neuronal survival [65].

### 4.8. Comparison Between miR-21 and Other miRNAs in SCI

In addition to miR-21, several other miRNAs, such as miR-124, miR-133b, miR-146a, miR-9, miR-182, miR-138, and miR-20a, play significant roles in SCI pathophysiology, influencing distinct aspects of the post-injury and recovery processes.

For instance, miR-124 acts as an anti-inflammatory regulator by promoting the polarization of microglia toward an anti-inflammatory (M2-like) phenotype, reducing tissue damage and creating a neuroprotective environment [100].

MiR-133b stands out for its regenerative role, promoting axonal outgrowth and functional recovery through pathways like ERK1/2 and PI3K/AKT, in stark contrast to the inhibitory effects of miR-21 [101]. MiR-146a, although similar to miR-21 in modulating inflammation, has a protective effect by targeting the NF-κB pathway to attenuate chronic inflammation, thereby minimizing neuronal damage [102].

MiR-9 supports neurogenesis and regulates inflammation through targets such as REST and SOCS3, fostering neural plasticity and regeneration rather than contributing to inhibitory mechanisms like miR-21 [103].

MiR-182 improves SCI outcomes by inhibiting apoptosis and the inflammatory response through the blockade of the IKKβ/NF-κB pathway, highlighting its therapeutic potential [104]. MiR-138 regulates axonal regeneration through interaction with SIRT1, a histone deacetylase, influencing neuronal growth and repair mechanisms [105].

MiR-20a, on the other hand, is associated with negative regulation of neuronal survival, as its overexpression in damaged spinal cord tissues contributes to sustained motor neuron degeneration [106].

While many of these miRNAs play important roles in the repair processes following SCI, miR-21 stands out as a potential therapeutic target due to its significant involvement in multiple injury-related pathways. Unlike other miRNAs that may have a more specialized role (e.g., miR-124 in inflammation or miR-133b in regeneration), miR-21 regulates many processes, including glial activation, scar formation, and immune response. This broad influence makes it a promising candidate for therapeutic modulation, as its inhibition or fine-tuning could provide more comprehensive control over the pathological cascades triggered by SCI.

Although limited information is available about miR-21 in the context of clinical trials, Tigchelaar et al. have identified, using next-generation sequencing, several mirRNAs associated with the severity of lesions in cerebrospinal fluid (CSF) and serum of patients with acute SCI [107].

Their study has shown that the best mirRNAs associated with the severity of the damage are miR-9-5p, along with miR-3p, miR-320a, miR-769, miR-9-3p, miR-219-2-3p, miR-432-5p, miR-128-3p, and miR-323a-3p [107].

As previously demonstrated, the identification of specific mirRNAs associated with the severity of SCI provides important insights into the molecular landscape of acute SCI, suggesting their potential as diagnostic biomarkers and therapeutic targets. Further research on the role of these miRNAs could lead to the development of new interventions aimed at modulating their expression, ultimately improving recovery outcomes for patients with SCI.

## 5. Conclusions and Future Perspectives

SCI is associated with a varying combination of symptoms including inflammatory responses, apoptosis, and dysregulation in motor functions. The physiology of the disease is not fully understood, but a complex interaction of several mechanisms is assumed to contribute to the progression of the trauma. MiR-21 is a short, single-stranded, and non-coding RNA involved in the post-transcriptional regulation of gene expression by inhibiting the translation and degradation of mRNAs. Studies have demonstrated an overexpression of miR-21 after SCI. This overexpression is often linked to various protective effects and modulates the signaling pathways involved in inflammation and cell survival. MiR-21 therefore represents both a diagnostic and a therapeutic challenge. A significant upregulation of miR-21 was found in SCI groups compared to the control group. When summarizing the data from the SCI and the control group, a positive correlation was found between miR-21 expression and the SCI, so an increased expression level was associated with a milder disease development.

MiR-21 has already been described in connection with anti-inflammatory properties, neuropathic processes, and the disruption of immune barriers by regulating the expression of pro-inflammatory receptors and mediators as well as influencing the differentiation of macrophages to provide an ideal microenvironment for the recovery of neurons. Knocking down miR-21 could, therefore, lead to a predisposition for excessive inflammatory reactions and thus contribute to the worsening of the spinal cord recovery process. Moreover, miR-21 exerts anti-apoptotic effects by targeting genes such as PDCD4 and PTEN, leading to reduced neuronal cell death. Studies have shown that increasing miR-21 levels can enhance neuronal survival in both in vitro and in vivo models of SCI. The proliferation and differentiation of NSCs are dramatically affected by miR-21. MiR-21 stimulates NSC proliferation and their differentiation into neurons and oligodendrocytes, which are necessary for efficient axonal regeneration, via stimulating AKT and GSK-3β t signaling pathways. Evidence illustrates that miR-21 can promote neuroregeneration, remyelination, and regulation of angiogenesis ultimately leading to functional recovery. However, a better understanding of the interaction between miR-21 and SCI may reveal therapeutic targets in future studies. Altered miRNA expression profiles are seen clearly in SCI. This makes it clear that understanding the function of miRNAs could enable potential therapeutic use with comprehensive significance. The collective results indicate the involvement of miR-21 in the pathophysiology of SCI. However, we must take into consideration that miR-21 is a versatile miRNA as its expression has been observed in various tissues and organs. Its upregulation is associated with a wide range of diseases, such as SCI but also cancer, fibrosis, and cardiovascular disorders [59,108,109]. Given its widespread functionality and role in modulating critical cellular processes like inflammation and apoptosis, its potential as a therapeutic target is substantial. Since miR-21 plays different roles in different tissues and diseases, a more nuanced approach could involve tailoring its modulation based on the specific context. The timing of intervention, whether to inhibit or activate miR-21, could also be crucial in achieving the desired therapeutic effect. Thus, future therapeutic strategies should focus on a combination of tissue-specific delivery, timing of miR-21 modulation, and potential combination with other treatments.

However, additional studies are required to clarify the precise regulatory networks involving miR-21, as well as the interaction with other miRNAs and signaling pathways.

## Figures and Tables

**Table 1:** Summary of the pre-clinical studies of miR-21′s role in neuroprotection and modulation of inflammation after SCI.

Description of the Study	Groups	Objective	Findings	Effect	Anesthesia/Injury Location	Reference
In vivo study Male C57BL/6 mice, n = 84	-Sham group with laminectomy -SCI group -SCI+ antagomir-21(50 μL/day, 100 nmol/mL) -SCI + antagomir-21-NC (50 μL/day, 100 nmol/mL)	Investigating miR-21-5p’s role in scar formation after spinal injury	miR-21-5p enhances TGF-β1’s activation of spinal fibroblasts, leading to fibrotic scar formation after SCI by targeting Smad7. Suppressing miR-21-5p improves motor function recovery.	-Antagomir-21 ↑ brain-derived neurotrophic factor (BDNF), ↑NGF -miR-21-5p ↑TGF-β1 ↓Smad7 ↓Scare formation	3% pentobarbital g/kg)/T8–T10	[60]
In vitro study with primary spinal fibroblasts	Scratch damage and negative control groups	Identify the role of miR-21a-5p in regulating spinal fibroblasts after mechanical trauma	The expression of miR-21a-5p was higher in spinal fibroblasts after scratch damage	miR-21a-5p mimic: promoted fibrogenic activity, enhancing proliferation and attenuating apoptosis in spinal fibroblasts	/	[61]
In vivo study Male Sprague-Dawley rats (n = 75), 300–330 g	-Sham (n =25) -Mild (n = 25) -Severe (n = 25)	Examine alterations in the degree of transcription of two microRNAs (miR-21 and -223) following TSCI at 4 h and 1, 3 and 7 days	No differences in the level of miR-21 expression were found at 4 h post-lesion between the three experimental groups, whereas such differences were significant at all the other time points	No compound administered	3% isoflurane and 2.5% isoflurane, ninth thoracic vertebra (T9)	[62]
In vivo study Female C57BL/6 mice, n = 40	-Sham group with laminectomy -SCI+ SCI group -miR-21 KD vector group -SCI + NC	Testing if reducing miR-21 encourages nerve repair and function	Knockdown of miR-21 improves motor function recovery and reduces inflammation by decreasing TGF-β1, TNF-α, IL-1β	-miR-21 KD ↑BDNF ↓TNFα, ↓TGF-β, ↓IL-1β, ↓p- Protein kinase B (AKT), T-AKT	3% pentobarbital (30 mg/kg, i.p.)/T9–T10	[63]
In vitro study with rat neuronal spinal cord cells	Control and H_2_O_2_ groups	Examine the role of miR-146a, miR-21, and miR-150 during H_2_O_2_ stimulation in rat neuronal spinal cord cells.	miR-146a, miR-21 and miR-150 expression was upregulated during H_2_O_2_ treatment	**/**	/	[64]
In vivo study Male Sprague-Dawley rat, n = 48/180–220 g	-Sham group with laminectomy -SCI group -SCI+ LV-miR21 -SCI+ Laser capture- negative control (LV-NC)	Exploring miR-21’s role in reducing neuron death post-spinal injury.	miR-21 improves neuronal survival and promotes functional recovery after injury. by reducing apoptosis through the miR-21/PDCD4/Casp-3 pathway.	-miR-21 ↓PDCD4/Casp-3 ↑NeuN	10% chloral hydrate (3 mg/kg)/T10	[65]
In vivo study Male Wistar rats, n = 43/250 g	-Sham with just surgical procedure -Control group (vehicles+ SCI) -Pre-miRNA-21+ SCI -Control vectors +SCI	Testing whether miR-21 overexpression protects spinal cells from damage.	Overexpression of miR-21 reduced apoptosis and improved motor function after SCI by inhibiting pro-apoptotic proteins such as Casp-3, FasL, and PDCD4.	-pre-miRNA-21 ↑miR-21, ↓Casp-3, ↓FasL, ↓PDCD4,	10% chloral hydrate (300 mg/kg)/L4-L6)	[66]
In vivo study C57BL/6 mice, n = NA	-Wild-Type (WT) Mice -Transgenic Reverse Orientation Splice Acceptor (ROSA)-miR21 -Transgenic ROSA-MSP	Studying how miR-21 impacts the role of astrocytes after injury.	Overexpression of miR-21 after SCI, leading to a reduced hypertrophic response and enhanced axon density at the lesion site.	-Gene Control MicroRNA (GCMIR) ↓GFAP, ↓Vimentin -NO EFFECT Ki67, cleaved Casp-3	inhalation of 2.5% isoflurane/T11	[68]
In vivo study Male C57BL/6 mice, n = 96	-Sham -Negative control TSCI + NC -TSCI + antagomir-21 -TSCI + antagomir-21+ *Cntfr* α siRNA	Assessing how miR-21 impacts reactive astrocyte formation and function.	miR-21a-5p promotes inflammation by transforming naïve astrocytes into neurotoxic reactive astrocytes (A1s) following TSCI through inhibition of the CNTF/STAT3/Nkrf pathway. CNTF inhibits A1 polarization by activating the STAT3 signaling pathway.	-Antagomir-21 ↑ S100a10, ↑ Cntfr α, ↑ p-STAT3/STAT3 ↓C3, ↓ Serpin Family G Member 1 (Serping1), ↓histocompatibility 2, D region locus 1(H2-D1)	3% pentobarbital (30 mg/kg, i.p.)/T8-T10	[69]
In vivo study Female C57BL/6 mice, n = 12/25–33 g	-Control -Abdominal Aortic occlusion	Investigating miR-21’s role in astrocyte activity during spinal injury recovery.	Silencing miR-21 promotes astrocyte polarization to the neuroprotective A2 phenotype, enhances synapse formation and nerve growth during ISCI, and targets glypican 6 via the STAT3 pathway.	NA	chloral hydrate (400 mg/kg)/(L4-L6)	[70]
In vivo study Male C57BL/6 mice, n = 52	-Sham group with laminectomy -SCI+ agomir-21 -SCI+ antagomir-21 -SCI+ NC	Examining miR-21’s effects on cell growth, cell death, and inflammation.	miR-21 is upregulated after SCI, promoting neuronal recovery by modulating astrocyte proliferation, apoptosis, and secretion via the TGF-β/PI3K/Akt/Mammalian target of rapamycin (mTOR) pathway.	-Agomir-21 ↑BDNF, ↑NGF, ↑GFAP, ↑ Chondroitin sulfate proteoglycans (CSPGs), ↑ Marker of proliferation Kiel 67 (Ki67), ↑p-AKT/AKT -Antagomir-21 ↓p-mTOR, ↓Ki67, ↓ B-cell lymphoma 2/Bcl-2-associated X protein (Bcl2/Bax)	10% chloral hydrate (3 mg/kg)/T8–T10	[71]
In vivo study Male Sprague-Dawley Rats, n = 24	-Sham group with laminectomy -SCI Group -SCI+ miR-21 Inhibitor	Looking at how miR-21 inhibitor influences inflammation after injury.	The inhibition of miR-21 reduced the expression of inflammatory factors iNOS and TNF-α and downregulated the IL-6R/Janus tyrosine kinase (JAK)-STAT signaling pathway after SCI, leading to improved recovery of locomotor function.	-miR-21 inhibitor ↓IL-6R, ↓JAK2, ↓STAT, ↓iNOS, ↓TNF-α	chloral hydrate (280 mg/kg)/T9-T11	[72]
In vivo study Female Sprague-Dawley rats, n = 150/200–250 g	-Sham control with laminectomy -SCI group	Studying miR-21 levels after injury and how they affect specific target genes such as PDCD4 and Phosphatase and tensin homolog (PTEN)	miRNA-21 expression levels decreased at 4 h, 8 h, and 1 day post-injury but increased at 3 and 7 days. Overexpression of miR-21 promotes neurite outgrowth by downregulating the pro-apoptotic protein PDCD4.	-After injury ↓PDCD4, ↓PTEN	1% pentobarbital sodium ip (50 mg/kg)/T9	[73]
In vivo study Sprague–Dawley rats, n = 56/180–220 g	-Negative control group received NC antagomir (1 μL/h, 20 nmol/mL) -Antagomir-21 group received antagomir-21 (1 μL/h, 20 nmol/mL) for 3 days	Investigating how miR-21 regulates apoptosis and affects recovery after spinal trauma.	miR-21 was upregulated after SCI in rats, and its knockdown worsened motor function recovery, increased lesion size, and elevated apoptosis by upregulating pro-apoptotic genes such as FasL and PTEN.	-Antagomir-21 ↑FasL, ↑PTEN,	10% chloral hydrate (10 mg/kg)/T10	[74]
In vivo study Sprague-Dawley rats, n = NA	-Sham control with laminectomy -SCI+ Exosomes—Scramble -SCI+ Exosomes -miR-21 -SCI+ Exosomes—PTEN siRNA:	Exploring how exosome-delivered miR-21 impacts movement recovery and cell death regulation after spinal injury.	miR-21 derived from mesenchymal stem cell (MSC) exosomes inhibits neuron cell death in SCI by modulating the expression of PTEN and PDCD4, promoting neuronal survival and differentiation.	-Exo-miR-21 ↓Casp-3, ↓PTEN, ↓PDCD4	10% chloral hydrate in saline (0.33 mL/kg)/T9-10	[75]

**Table 2:** Experimental Design and miR-21 Dosing Protocols in in vivo SCI studies.

SCI Model	SCI Level	Severity	Drug Dose	Treatment Duration	Administration Route	Mir-21 > 0.05	Reference
Contusion	T8–T10	Moderate	-Antagomir-21 (50 μL/day, 100 nmol/mL) -miR-21 NC (50 μL/day, 100 nmol/mL)	3 days after SCI	Intrathecal injections	Yes	[60]
Contusion	T9–T10	Moderate	-miR-21-KD (1 × 10^7^ Transducing Units).	Single dose after injury	Subdural injection	Yes	[63]
Contusion	T10	Moderate	-LV/miR-21 (5 μL)	Not clearly reported	Injection in rostral and caudal areas around the lesion	Yes	[65]
Ischemia	L4-L6	Not available	-LV/pre-miRNA-21 (NA)	5 days before SCI	Intrathecal injection	Yes	[66]
Contusion	T11	Severe	-Transgenic mice with overexpressed miR-21	Not available	Not available	Yes	[68]
Percussion	T8-T10	Not available	-Antagomir-21 (2.5 μL, 2 μmol/mL) -Antagomir-21 + Cntfr α siRNA 0.5 nmol (1 μL, 0.5 μmol/mL)	-Antagomir -21 three days 0, 1, 2 after SCI -Antagomir -21 + Cntfr α siRNA one time day 0	Intrathecal injection	Yes	[69]
Acute ischemic	L4-L6	Not available	Not available	Not available	Not available	Yes	[70]
Contusion	T8–T10	Moderate	-Antagomir-21 (50 μL/day, 100 nmol/mL) -Agomir-21 (50 μL/day, 100 nmol/mL)	3 days after SCI	Intrathecal injections	Yes	[71]
Contusion	T9-T11	Moderate	-miR-21 inhibitor (NA)	Single dose after injury	Intrathecal injection	Yes	[72]
Contusion	T9	Moderate	Not available	Not available	Not available	Yes	[73]
Contusion	T10	Moderate	-Antagomir-21 (1 µL/h, 20 nmol/mL)	3 days	Intrathecal injection	Yes	[74]
Contusion	T9-10	Moderate	-Exosomes with miR-21 (NA) -Exosomes with PTEN siRNA (NA)	Not available	Not available	No	[75]

Agomir-21: synthetic molecule designed to mimic miR-21; Antagomir-21: synthetic oligonucleotide designed to inhibit miR-21 functions.

## Data Availability

Not applicable.

## Abbreviations

miR-21	microRNA-21
SCI	Spinal cord injury
PTEN	Phosphatase and tensin homolog
PDCD4	Programmed cell death protein 4
TNF-α	Tumor necrosis factor-α
NMDA	N-methyl-D-aspartate
AMPA	alpha-amino-3-hydroxy-5-methyl-4-isooxazole-propionic acid
ROS	Reactive oxygen species
RNS	Reactive nitrogen species
NF-κB	Nuclear factor-kappa B
NSCs	Neuronal stem cells
eNOS	Endothelial nitric oxide synthase
iNOS	Inducible nitric oxide synthase
BDNF	Brain-derived neurotrophic factor
NGF	Nerve growth factor
Smad7	Mothers against decapentaplegic homolog 7
NeuN	Neuron specific nuclear protein
AKT	Protein kinase B
Ki67	Marker of proliferation Kiel 67
mTOR	Mammalian target of rapamycin
Bcl2	Apoptosis regulator Bcl2
Bax	Bcl-2–associated X protein
JAK	Janus tyrosine kinase
STAT	Signal transducer and activator of transcription
H2-D1	histocompatibility 2, D region locus 1
Serping 1	Serpin family G member 1
TSCI	Traumatic spinal cord injury
CSPGs	Chondroitin sulfate proteoglycans
LV-NC	Laser capture- negative control
Bad	BCL2 associated agonist of cell death
ROR2	Tyrosine-protein kinase transmembrane receptor 2
CNTFRα	Ciliary neurotrophic factor receptor α
ROSA	Reverse Orientation Splice Acceptor
GCMIR	Gene Control MicroRNA
PLAG1	Pleomorphic Adenoma Gene 1
KRIT1	Krev Interaction Trapped 1
SOD3	Superoxide Dismutase 3
TrkB	tropomyosin receptor kinase B
JNK	c-Jun N-terminal Kinase
GFAP	Glial Fibrillary Acidic Protein
PI3K	Phosphoinositide 3-Kinase
TGF-β	Transforming Growth Factor-β
FASR	Fas Receptor
PCNA	Proliferating Cell Nuclear Antigen
Serping1	Serpin Family G Member 1
Frizzled	represent a family receptors coupled to Wnt signaling
Casp-3	Caspase-3
Casp-8	Caspase-8
